# Glomerular filtration rate estimated by the Chronic Kidney Disease Epidemiology Collaboration (CKD-EPI) equation in type 1 diabetes based on genomic ancestry

**DOI:** 10.1186/s13098-020-00578-4

**Published:** 2020-08-15

**Authors:** Marcela Haas Pizarro, Deborah Conte Santos, Laura Gomes Nunes Melo, Bianca Senger Vasconcelos Barros, Luiza Harcar Muniz, Luís Cristóvão Porto, Dayse Aparecida Silva, Rachel Bregman, Marilia Brito Gomes

**Affiliations:** 1 Department of Internal Medicine, Diabetes Unit, Rio de Janeiro State University (UERJ), Boulevard 28 de Setembro, 77- 3º andar - Vila Isabel, Rio de Janeiro, RJ CEP 20551-030 Brazil; 2 Department of Ophthalmology, Rio de Janeiro State University (UERJ), Rio de Janeiro, RJ, Brazil; 3 Histocompatibility and Cryopreservation Laboratory (HLA), Rio de Janeiro State University (UERJ), Rio de Janeiro, RJ Brazil; 4 DNA Diagnostic Laboratory (LDD), Rio de Janeiro State University (UERJ), Rio de Janeiro, RJ Brazil; 5 Department of Internal Medicine, Nephrology Unit, Rio de Janeiro State University (UERJ), Rio de Janeiro, RJ Brazil

**Keywords:** Chronic kidney disease, Genomic ancestry, Self-reported color-race, Type 1 diabetes, Chronic Kidney Disease Epidemiology Collaboration (CKD-EPI) equation

## Abstract

**Background:**

Black individuals have a great risk of developing chronic kidney disease (CKD) that is associated with high morbimortality, so it is important to classify them into the correct renal function group. Some equations used to estimate glomerular filtration rate (eGFR) divide patients only into two categories: African Americans and non-African Americans. The CKD-EPI equation was tested only in African Americans, and not Black patients from other regions, and takes into consideration self-reported color-race instead of genomic ancestry (GA) to determine the use of the ethnic correction factor. So far, this equation has not been evaluated in admixed populations, such as the Brazilian, using the percentage of GA to decide to apply the correction factor. The purpose of our study was to compare, in patients with type 1 diabetes (T1D), the eGFR calculated without the use of the correction factor, with the values obtained using the correction factor in patients presenting 50% or more of African GA.

**Methods:**

This cross-sectional, multicenter study enrolled 1279 patients from all geographic regions of Brazil. The CKD-EPI equation was used and CKD was defined as eGFR < 60 ml/min. GA were inferred using a panel of 46 AIM-INDEL, afterwards patients presenting an African GA ≥ 50% were selected.

**Results:**

Initially, all patients with African GA ≥ 50% (n = 85) were considered as non-African Americans when calculating the eGFR and afterwards the ethnic correction factor was applied to recalculate the eGFR. CKD was present in 23 patients and 56.5% of them were redefined as having normal renal function after using the correction factor, mainly women [11 of the 13 patients (84.6%)], with GFR between 52–59.3 ml/min.

**Conclusions:**

More than half of the patients in the study were reclassified to a normal renal function group, showing that GA may be an important tool to decide between the use of the ethnic correction factor in the CKD-EPI equation in a highly admixed population of patients with T1D. A large-scale study involving GA and eGFR in comparison to reference methods should be conducted to better establish whether or not the ethnic correction factor should be used in highly admixed populations.

## Background

African Americans and Black individuals from other regions have a greater burden of chronic kidney disease (CKD) than whites, and that is associated with high morbimortality rates. They present a higher proportion of muscle mass compared to Caucasians and Asians [1], resulting in a higher creatinine generation that should be considered when estimating the glomerular filtration rate (eGFR). So far, the two most frequently equations used worldwide to estimate GFR, the Modification of Diet in Renal Disease (MDRD) and the Chronic Kidney Disease Epidemiology Collaboration (CKD-EPI) equation, were developed based on populations with low percentages of non-Caucasian individuals, so their accuracy in other populations is questionable [2]. These equations were tested only in African Americans, and not in Black patients from other regions such as Africa or South America. The use of the correction factor takes into account ethnicity as a dichotomous variable: African Americans or non-African Americans. Additionally, the aforementioned equations generally take into consideration self-reported color-race to use or not the ethnic correction factor.

The Brazilian population is formed by 3 main ancestral contributions: Europeans (EUR), Africans (AFR) and Native Amerindians (NAM) and has a great heterogeneity due to five centuries of interethnic miscegenation among these three groups. The Brazilian census classifies self-reported color-race into 5 categories: White, Black, Brown (“parda”), Asian (“amarela”) and Indigenous (“indígena”) [3]. However, it is important to emphasize that recent studies have shown that self-reported color-race is not a reliable tool to evaluate ethnicity in a highly admixed population such as the one found in Brazil [4, 5]. Nowadays, the development of new methodologies such as the inference of genomic ancestry (GA) can be used to better stratify patients from highly admixed populations, with a lower probability of misclassification. A more precise stratification has a better chance of identifying groups that have a higher risk of developing CKD. Therefore, we used autosomal Ancestry Informative Markers (AIMs) to evaluate the percentage of GA and to better stratify our patients.

However, despite having a heterogenous population, the CKD-EPI equation is still largely used in Brazil to estimate the GFR and the Black patients are classified as African Americans, despite having different percentages of AFR ancestry.

This study divided patients according to levels of GA, instead of self-reported color-race and only used the ethnic correction factor in patients with an AFR ancestry of 50% or more. The purpose of this study was to compare, among patients with type 1 diabetes (T1D), the eGFR calculated without the use of the correction factor for Black individuals, with the values obtained using the correction factor in those presenting 50% or more of AFR GA.

## Methods

This is a cross-sectional, observational, multicenter study. Initially, 1279 adults with T1D from all geographical areas of Brazil were included. Patients and methods were described previously [6]. Briefly, all patients were diagnosed with T1D based on the presence of classic clinical presentation at the moment of the diagnosis, such as polyuria, weight loss, polydipsia, and the need for continuous insulin use since the diagnosis. These patients were followed in secondary or tertiary centers, from the National Brazilian Health Care System (SUS) by an endocrinologist. Inclusion criteria were: patients older than 19 years of age and medical follow-up for at least 6 months at the respective center. Exclusion criteria were: pregnancy or lactation at the moment of inclusion, history of renal transplant, and acute infection or ketoacidosis in the three months before the recruitment. All the variables were obtained using a questionnaire during a clinical visit that was also described previously [6] and included in the present study such as gender, age, duration of diabetes (years) and self-reported-color-race [White, Black, Brown (“parda”), Asian (“amarela”) and Indigenous (“indígena”)] [3].

Creatinine was measured using a colorimetric assay kit (Biosystems, that is corrected for standardized creatinine assay by mass spectrometry). Renal function was estimated by the CKD-EPI equation [7].

We used the commercial kit SP QIA symphony by automation with QIA symphony equipment, following manufacturer's instructions (Qiagen, USA) to extract Genomic DNA from peripheral blood. A panel of 46 AIM-INDEL was used to infer the global and individual GA according to the protocol described by Pereira et al. [8]. Genotyping was done by multiplex PCR followed by capillary electrophoresis with the ABI 3500 sequencer. The software Gene Mapper V.4.1 (Life Technologies, USA) was used for Allele naming and results were compared for consistency. We used the Structure V.2.3.3 software to estimate ancestry and the HGDP-CEHP diversity panel (Sub-Set H952) as reference date of ancestral populations. Structure ran with 100,000 burning steps followed by 100,000 Markov Chain Monte Carlo (MCMC) interactions using the "Admixture model" default correlating allele frequencies and the number of populations (K = 3), designated as EUR, AFR and NAM. GA was expressed as percentages, with the sum of EUR, AFR and NAM equaling 100%.

Initially, all patients were considered as non-African Americans when calculating the eGFR, even those that self-reported themselves as Black. Patients were classified considering their renal function into two groups: normal renal function group (eGFR ≥ 60 ml/min) and CKD group (eGFR < 60 ml/min). Afterwards, the ethnic correction factor was applied to recalculate eGFR in those who presented an AFR ancestry of 50% or higher.

The study was approved by the ethics committee of Pedro Ernesto University Hospital (State University of Rio de Janeiro), and by the local ethics committee of each center. All participants signed the informed consent form.

## Statistical analysis

Continuous variables are presented as the medians [interquartile range] and frequencies and percentages were used to present categorical variables. Mann–Whitney test was used for comparison of continuous variables and Fisher exact test was used for comparison between categorical variables. All analyses were performed using the Statistical Package for the Social Sciences (SPSS version 17.0, SPSS, Inc., Chicago, Illinois, USA). A two-sided *p* value less than 0.05 was considered significant.

## Results

African GA ≥ 50% was present in 85 patients. Seven (8.2%) self-reported themselves as White, 40 (47.1%) as Black, 37 (43.5%) as Brown and one (1.2%) as Indigenous. CKD was present in 23 patients (27%), the others (73%) presented a normal renal function, that was defined as eGFR ≥ 60 ml/min (Table 1). In Table 1 we compared these patients with the other 1194 patients that had an AFR < 50%. The distribution into groups of renal function was very similar between both groups, with 28.6% of patients with AFR < 50% with CKD and 27% of those with AFR ≥ 50% with CKD. The median of AFR, EUR, and NAM ancestry was also described in this table and compared between the 2 groups.

**Table 1:** Demographic data of the studied population

	Patients with AFR ancestry < 50%	Patients with AFR ancestry ≥ 50%	*p* value
n	1194	85	
Gender, n (% female)	691 (57.9)	48 (56.5)	0.8
Age, years	32 [16]	31 [14]	0.84
Duration of diabetes, (years)	16 [12]	13 [9.5]	0.09
Weight (kg)	66 [17.1]	67.5 [21.2]	0.39
Self-reported color-race, n (%)			< 0.001
White	710 (59.5)	7 (8.2)	
Black	68 (5.7)	40 (47.1)	
Brown	397 (33.2)	37 (43.5)	
Asian	12 (1)	0	
Indigenous	7 (0.6)	1 (1.2)	
Geographic region, n (%)			< 0.001
Southeast	595 (49.8)	31 (36.5)	
South	176 (14.7)	7 (8.2)	
North/northeast	286 (24)	40 (47.1)	
Mid-West	137 (11.5)	7 (8.2)	
Use of renin-agiotensin system blockers (n, %)	378 (31.9%)	40 (47%)	0.004
Statin use (n, %)	320 (26.8%)	21 (24.7%)	0.673
Genomic ancestry, %			
African	14.0 [20.6]	61 [12]	< 0.001
European	69.8 [28.7]	27 [17]	< 0.001
Amerindian	10 [16.2]	9 [8.4]	0.367
Renal function, n (%)			0.44
Normal (eGFR ≥ 60 ml/min)	853 (71.4)	62 (73)	
CKD	341 (28.6)	23 (27)	

**Fig. 1:**
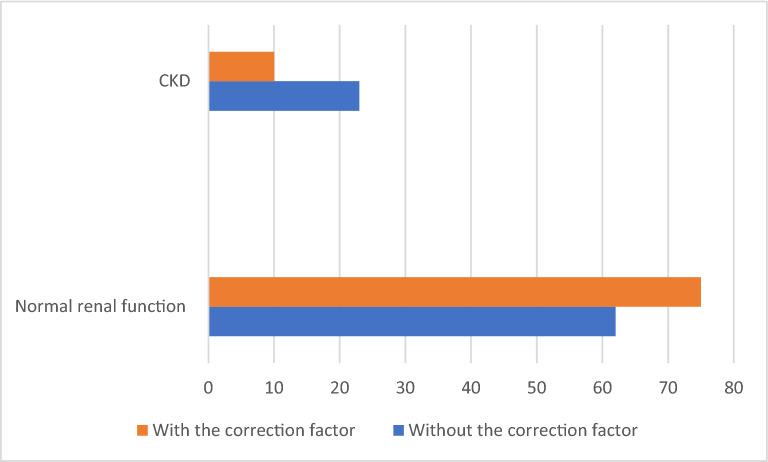
Renal function with and without the use of the correction factor. Data are presented as percentage. *CKD* Chronic Kidney Disease

The ethnic correction factor was used to recalculate the eGFR in those patients with an AFR ancestry ≥ 50%, resulting in 13 patients (56.5%) with an eGFR ≥ 60 ml/min and thus reclassified as normal renal function group (Fig. 1). The reclassification was more frequent in female than male patients [11 (84.6%) vs 2 (15.4%)]; *p* = 0.02, respectively, in patients with lower GFR [57.9 (52–59.3) vs 85 (12.4–138 ml/min); *p* < 0.001], respectively, and had a tendency to be more frequent in older patients (39.3 ± 12.6 vs 32.2 ± 9.4 years; *p* = 0.05), respectively. No difference was noted in the diabetes duration (18.9 ± 11.9 vs 14.9 ± 6.0 years, *p* = 0.8), weight [67.4 (23.0) vs 67.5 (19.8) kg, *p* = 0.6), percentage of AFR GA [59.65% (13.2) vs 64.1% (6.3), *p* = 012], and the use of renin-angiotensin system inhibitors [6 (46.1%) vs 34 (47.2%), *p* = 0.9] between patients reclassified in comparison to patients who were not reclassified, respectively.

## Discussion

This study is, to the best of our knowledge, the first to consider GA instead of self-reported color-race to choose between the use or not of the ethnic correction factor in the CKD-EPI equation in a highly admixed population of patients with T1D. We observed that the use of the correction factor in patients with AFR GA ≥ 50% resulted in the reclassification of 56.5% of the patients to a normal renal function stage, mainly females with an eGFR between 50–59 ml/min.

The evaluation of GFR by reference methods such as inulin or other exogenous biomarkers are cumbersome and expensive [9]. Estimated GFR, using endogenous biomarkers like creatinine and cystatine C, is a useful tool in routine clinical practice because it allows the stratification of our patients quickly and with a reasonable precision. So far, different equations to estimate GFR have been proposed, such as Cockcroft and Gault and Modification of Diet in Renal Disease (MDRD). In general, these equations were derived from small samples, mainly formed by Caucasians [10]. In 2009, the CKD-EPI equation was created based in a large database pooled from ten studies comprising 8254 participants, covering a more diverse population than that of the MDRD study, with 32% of individuals reporting themselves as African Americans [7]. Both equations, the MDRD and CKD-EPI, were the first to introduce an ethnic correction factor based on self-reported color-race or ethnicity obtained in medical records, resulting in an increase of eGFR. The use of this correction factor is still controversial. In general, studies from other African populations from South Africa [11], Ghana [12], Congo [13] and studies with Black patients from Europe [14] and Indian patients [15] have concluded that the use of the correction factor seems to be inadequate, and that it is necessary to have a specific correction factor for each studied African population [16]. The aforementioned studies have compared the eGFR with the renal clearance determined by the reference methods and have included different individuals from the general population with comorbidities that are risk factors for the development of CKD, such as diabetes, hypertension and HIV.

Although it is beyond the scope of the present study to discuss ethnicity as a social, economic and cultural construct, it is important to emphasize that recently it was argued that the determination of the individual GA is a better tool to stratify our patients in clinical studies [10].

The present study, at first, calculated eGFR considering all patients as non-African Americans. Afterwards, the eGFR was calculated using the ethnic correction factor for those with an AFR GA ≥ 50%. It is important to note that a low percentage of AFR ancestry, even in those that self-reported themselves as Blacks, is observed in the Brazilian general population [4] [5] as well as in a population of patients with T1D [17], revealing that self-reported color-race is not a good tool to stratify patients according to ethnic groups in highly admixed populations, such as the Brazilian. The median AFR ancestry of our baseline Black self-reported population is lower than that found in African Americans, 43.7% [18] vs 82% [19], respectively, reflecting the high degree of miscegenation of our population. It is important to note that the study mentioned above, conducted in the USA, was performed in the general population, with only 30% of patients from their sample with diabetes.

In this same study, Udler et al. [19] used GA to recalculate eGFR by the CKD-EPI equation in those patients with an AFR ancestry ≥ 50%. They showed that 1% of African Americans that had an AFR ancestry of less than 50% were reclassified to a higher (worse) CKD stage, since they removed the ethnic correction factor. The study also evaluated Hispanic/Latin Americans. Those with an AFR ancestry ≥ 50% had their eGFR recalculated with the use of the ethnic correction factor and 4.3% of them were reclassified to a lower (less severe) CKD stage. In our population, from those with an AFR ancestry ≥ 50, 56.5% were reclassified into the normal renal function group, suggesting that there is a significant difference if the correction factor is used. However, it is important to emphasize that the cutoff point ≥ 50% of African GA was arbitrarily chosen in both studies.

Considering the different levels of eGFR, a study conducted in healthy Black Africans, from Kenya without risk factors for CKD, showed that the CKD-EPI equation with the use of the ethnic correction factor was better for the classification of these patients as having a normal eGFR compared to other equations [20]. However, a study conducted in Black Africans with HIV showed that the CKD-EPI equation without the use of the ethnic correction factor was more accurate in estimating GFR that were ≤ 90 ml/min [21]. Conversely, the use of the correction factor did not change the accuracy of the eGFR > 90 ml/min. Therefore, there is not a consensus about the use of the ethnic correction factor in the entire Black African population.

Since patients that where reclassified to the normal renal function group were those that had an eGFR of 50–59 ml/min, we probably should monitor them closely and look for other indications as to which renal function group they truly belong. The patients with an AFR ancestry of 50% or higher had their eGFR increased by the factor 1.159, so patients with lower eGFR despite having an increase in the eGFR, the increase was not sufficient to change their renal function group.

A study conducted in Brazil, involving 244 individuals, analyzed the eGFR by different equations, including CKD-EPI and compared it to the GFR determined by the plasma clearance of iohexol. They concluded that the CKD-EPI equation without the use of the ethnic correction factor was not more accurate in estimating the GFR then the equation with the use of the correction factor [22]. However, it is important to note that the use of the ethnic correction factor in this study was based in self-reported color-race, and not in GA.

An important strength of our study is the population-based ascertainment of diabetes cases in a large sample of Brazilian patients with T1D, from an admixed population, from all geographic regions of the country. All participating centers followed a uniform and standardized protocol and all the laboratory analyzes were performed in the same center. Similar to other epidemiologic studies, we used a uniform clinical definition of T1D.

Finally, our study has also some limitations that must be mentioned. The first was the sample characteristics. All patients lived in urban areas and received medical care in public health care centers by a specialist; thus, patients who rely on primary care settings and live in rural areas may not have been represented. However, the latter group of patients with T1D are the minority of those receiving treatment in Brazil. Second, the cutoff used for AFR GA was arbitrarily chosen because so far there are no cutoff values to determine the relationship between GA and ethnic groups. However, this value was used in another study conducted in African Americans [19]. Third, the GFR was estimated and was not measured by reference methods.

## Conclusions

Our study is the first to consider GA instead of self-reported color-race to decide between the use of the ethnic correction factor in the CKD-EPI equation in a highly admixed population of patients with T1D. Ideally, the eGFR equations should consider the GA instead of self-reported color-race in highly admixed populations, mainly in patients with GFR between 50 and 59 ml/min.

A large-scale study involving GA and eGFR in comparison to reference methods for determination of GFR should be conducted to better establish whether or not the ethnic correction factor should be used to obtain more precise eGFR values in highly admixed populations in routine clinical practice.

## Data Availability

The datasets used and/or analyzed during the current study are available from the corresponding author on reasonable request.

## Abbreviations

CKD: Chronic kidney disease
eGFR: Estimated glomerular filtration rate
CKD-EPI: Chronic Kidney Disease Epidemiology Collaboration study
GA: Genomic ancestry
T1D: Type 1 diabetes
EUR: Europeans
AFR: Africans
NAM: Native Amerindians
AIMs: Ancestry Informative Markers

## References

[1] Gallagher D, Visser M, De Meersman RE, Sepúlveda D, Baumgartner RN, Pierson RN (1985). Appendicular skeletal muscle mass: effects of age, gender, and ethnicity. J Appl Physiol.

[2] Delanaye P, Mariat C, Maillard N, Krzesinski JM, Cavalier E (2011). Are the creatinine-based equations accurate to estimate glomerular filtration rate in African American populations?. Clin J Am Soc Nephrol.

[3] IBGE (2008). Características étnico-raciais da população: um estudo das categorias de classificação de cor ou raça.

[4] Parra FC, Amado RC, Lambertucci JR, Rocha J, Antunes CM, Pena SD (2003). Color and genomic ancestry in Brazilians. Proc Natl Acad Sci U S A.

[5] Leite TK, Fonseca RM, de França NM, Parra EJ, Pereira RW (2011). Genomic ancestry, self-reported "color" and quantitative measures of skin pigmentation in Brazilian admixed siblings. PLoS ONE.

[6] Gomes MB, Negrato CA (2016). Adherence to insulin therapeutic regimens in patients with type 1 diabetes. A nationwide survey in Brazil. Diabetes Res Clin Pract.

[7] Levey AS, Stevens LA, Schmid CH, Zhang YL, Castro AF, Feldman HI (2009). A new equation to estimate glomerular filtration rate. Ann Intern Med.

[8] Pereira R, Phillips C, Pinto N, Santos C, dos Santos SE, Amorim A (2012). Straightforward inference of ancestry and admixture proportions through ancestry-informative insertion deletion multiplexing. PLoS ONE.

[9] Webster AC, Nagler EV, Morton RL, Masson P (2016). Chronic kidney disease. Lancet.

[10] Morris H, Mohan S (2020). Using race in the estimation of glomerular filtration rates: time for a reversal?. Curr Opin Nephrol Hypertens.

[11] van Deventer HE, George JA, Paiker JE, Becker PJ, Katz IJ (2008). Estimating glomerular filtration rate in black South Africans by use of the modification of diet in renal disease and Cockcroft-Gault equations. Clin Chem.

[12] Eastwood JB, Kerry SM, Plange-Rhule J, Micah FB, Antwi S, Boa FG (2010). Assessment of GFR by four methods in adults in Ashanti, Ghana: the need for an eGFR equation for lean African populations. Nephrol Dial Transplant.

[13] Bukabau JB, Sumaili EK, Cavalier E, Pottel H, Kifakiou B, Nkodila A (2018). Performance of glomerular filtration rate estimation equations in Congolese healthy adults: The inopportunity of the ethnic correction. PLoS ONE.

[14] Flamant M, Vidal-Petiot E, Metzger M, Haymann JP, Letavernier E, Delatour V (2013). Performance of GFR estimating equations in African Europeans: basis for a lower race-ethnicity factor than in African Americans. Am J Kidney Dis.

[15] Moodley N, Hariparshad S, Peer F, Gounden V (2018). Evaluation of the CKD-EPI creatinine based glomerular filtration rate estimating equation in Black African and Indian adults in KwaZulu-Natal. South Africa Clin Biochem.

[16] Stevens LA, Claybon MA, Schmid CH, Chen J, Horio M, Imai E (2011). Evaluation of the Chronic Kidney Disease Epidemiology Collaboration equation for estimating the glomerular filtration rate in multiple ethnicities. Kidney Int.

[17] Gomes MB, Gabrielli AB, Santos DC, Pizarro MH, Barros BSV, Negrato CA (2018). Self-reported color-race and genomic ancestry in an admixed population: a contribution of a nationwide survey in patients with type 1 diabetes in Brazil. Diabetes Res Clin Pract.

[18] Pizarro MH, Santos DC, Melo LGN, Barros BSV, Muniz LH, Porto LC (2019). Influence of genomic ancestry and self-reported color-race in CKD in a nationwide admixed sample of Brazilian patients with type 1 diabetes. Diabetes Metab Syndr Obes.

[19] Udler MS, Nadkarni GN, Belbin G, Lotay V, Wyatt C, Gottesman O (2015). Effect of genetic african ancestry on eGFR and kidney disease. J Am Soc Nephrol.

[20] Omuse G, Maina D, Mwangi J, Wambua C, Kanyua A, Kagotho E (2017). Comparison of equations for estimating glomerular filtration rate in screening for chronic kidney disease in asymptomatic black Africans: a cross sectional study. BMC Nephrol.

[21] Wyatt CM, Schwartz GJ, Owino Ong'or W, Abuya J, Abraham AG, Mboku C (2013). Estimating kidney function in HIV-infected adults in Kenya: comparison to a direct measure of glomerular filtration rate by iohexol clearance. PLoS ONE.

[22] Zanocco JA, Nishida SK, Passos MT, Pereira AR, Silva MS, Pereira AB (2012). Race adjustment for estimating glomerular filtration rate is not always necessary. Nephron Extra.

